# Hyperacute relapse of Lewis-Sumner syndrome during influenza A (H1N1) virus infection

**DOI:** 10.1186/s12883-020-02008-4

**Published:** 2020-11-24

**Authors:** Luís Ribeiro, Ana Monteiro, João Martins

**Affiliations:** 1 Department of Neurology, Unidade Local de Saúde de Matosinhos, Hospital Pedro Hispano, Sra. da Hora, Portugal; 2 Faculty of Medicine of University of Porto, Porto, Portugal; 3 MedicilLisboa, Lisboa, Portugal

**Keywords:** Lewis-Sumner syndrome, Influenza a virus, H1N1, Hyperacute relapse

## Abstract

**Background:**

Lewis-Sumner Syndrome (LSS) is considered an asymmetric sensory-motor variant of Chronic Inflammatory Demyelinating Polyneuropathy (CIDP), mostly affecting the limbs distally, with electrophysiological evidence of multifocal motor conduction blocks. Cranial nerve involvement is present in a minority. Various well-known infectious agents, directly or via the host’s immune responses, may trigger or exacerbate acute and chronic peripheral neuropathies, which may manifest clinically through a multitude of signs and symptoms.

**Case presentation:**

We present the case of a 57-year-old male with Lewis-Sumner Syndrome, whose clinical course was quite stable over many years. He developed severe hyperacute relapse of his neuropathic disease in the context of active pneumonia due to influenza A (H1N1) virus infection. During this exacerbation, besides the obvious worsening of the previous asymmetric limb involvement, the patient also manifested left peripheral facial palsy and dysphagia that rapidly evolved over minutes, mimicking a stroke. The patient also showed rapid recovery, with marked improvement of the acute neuropathic dysfunction, immediately after initiation of treatment with oseltamivir. Our hypothesis is that the direct modulation of Na + ion channel activity in the host’s peripheral nerve cell by H1N1 viral proteins could cause acute and potentially reversible dysfunction in the conduction of nerve action potentials. Direct viral neuritis could also have been the cause. Immunomodulatory agents, namely IVIg, were not administered due to the swift clinical improvement noticed in the following days.

**Conclusions:**

We aim to raise awareness of the possibility of atypical neurological presentations of viral infections, especially relevant in the context of the pandemic the world is now facing.

## Background

Lewis-Sumner Syndrome (LSS) is considered an asymmetric sensory-motor variant of Chronic Inflammatory Demyelinating Polyneuropathy (CIDP), affecting the limbs mostly distally, with electrophysiological evidence of multifocal motor conduction blocks. The symptoms usually begin in an upper limb, with hand and forearm weakness. Distal limb amyotrophy is present in about half the patients and cranial nerve involvement is present in a minority. The disease is chronically progressive in 70% of cases and relapsing-remitting in the remainder 30% [1].

Various well-known infectious agents, directly or via the host’s immune responses, may be the trigger of acute and chronic peripheral neuropathies, or of their clinical exacerbation, which may manifest through a multitude of signs and symptoms [2–5].

The 2009 pandemic influenza A (H1N1) virus infection is frequently associated with central and/or peripheral neurological signs and symptoms. The most common neurological manifestations include headache, numbness and paresthesia, acute neuropathies, drowsiness, epileptic seizures and coma. Diagnostic testing for H1N1 influenza virus is recommended in all patients with respiratory illness and neurological signs/symptoms [6, 7]. Despite its association with various subtypes of Guillain-Barré Syndrome, to our knowledge, there is no report of an hyperacute relapse of CIDP related to H1N1 virus.

## Case presentation

We present the case of a 57-year-old male fulfilling clinical and electrophysiological diagnostic criteria for Lewis-Sumner Syndrome beginning at the age of 43 years [8]. He had no family history of neuropathies nor other neurological disease. After an initial subacute onset, the clinical history had been slowly progressive, reaching a plateau of clinical disability that had been stable for the next 10 years. Antiganglioside antibodies (anti-GQ1b, anti-GD3 and anti-GM1) were negative. The disability consisted mainly of distal sensory-motor dysfunction of the right lower limb with foot drop (eversion and dorsiflexion plegia), despite the presence of multiple subclinical electrophysiological signs of an asymmetric multifocal demyelinating polyneuropathy with conduction blocks, affecting both upper and lower limbs, in the electromiographies with nerve conduction studies (NCS/EMG) performed over time. He scored 45/48 in the inflammatory Rasch-built Overall Disability Scale (iRODS) for immune-mediated peripheral neuropathies, translating very mild clinical disability [8, 9].

**Fig. 1:**
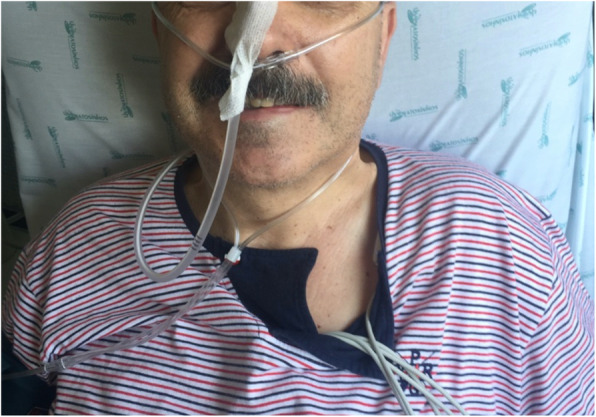
Peripheral left facial nerve palsy at admision

**Fig. 2:**
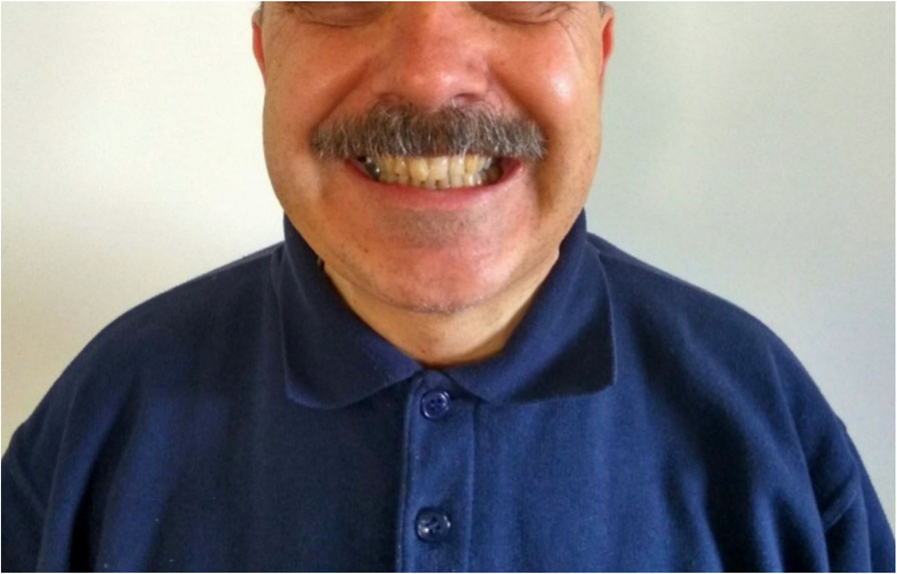
Almost complete recovery of the facial palsy after 2 weeks

In January 2019, in the context of severe pneumonia due to Influenza A (H1N1) virus infection, ongoing for 4 days, he developed hyperacute (onset in a few minutes) left peripheral facial palsy with prominent lower half predominance, dysphagia, dysphonia, cervical flexion paresis, sensory-motor dysfunction of the left arm and global worsening of his right leg motor deficit (now also affecting plantar flexion and knee extension), mimicking multifocal embolic and/or brainstem stroke (Fig. 1). He was rapidly admitted in the emergency room and received full stroke protocol, including urgent cranial computer tomography (CT) scan and angio-CT, without any evidence of acute brain lesions. After 72 h, brain magnetic resonance imaging (MRi) was carried out, definitively excluding acute vascular injuries. CSF analysis was unremarkable, revealing no albuminocytologic dissociation. NCS/EMG were performed 9 days after the onset of the acute neurological dysfunction, confirming the neuropathic nature and revealing a new relapse, with electrophysiological criteria for asymmetric CIDP with bilateral facial involvement, with markedly increased facial distal motor latencies (DML), worse on the left as show in Tables 1 and 2. The MRI images were reviewed and no signal changes of the cranial nerves were reported, although the absence of thin cuts of the infratentorial region limited cranial nerve assessment. He reached a minimum iRODS score of 07/48 during the peak of this relapse, indicating severe clinical disability. The patient was promptly started on oseltamivir on the first day of admission upon laboratorial confirmation of H1N1 virus RNA in the serum. Fortunately, improvement was noted soon after treatment initiation and the patient was able to eat small portions of pasty diet on day 2. The relapse episode swiftly evolved favorably in the following days almost returning to his basal condition after 6 days (Fig. 2). The patient greatly recovered of the acute neurological deficits presented at admission, even without the use of IVIg, steroids or other immunomodulatory therapies, which were not started due to the favorable clinical course. His iRODS score greatly improved to 42/48 at the 3rd month of follow-up, although not fully to the previous status, and remains stable after 1 year (as of January 2020).

**Table 1:** Motor Nerve Conduction Studies Before and After Influenza A Infection

	Before H1N1 infection				After H1N1 infection (after 9 days)			
Nerve	DML	AMP	CV	Δ AMP	DML	AMP	CV	Δ AMP
**Left Zygomatic**	NP	NP			12.80 ms^a^	0.24 mV		
**Right Zygomatic**	NP	NP			5.63 ms^a^	1.97 mV		
**Left Mandibular**	NP	NP			12.10 ms^a^	0.34 mV		
**Right Mandibular**	NP	NP			3.61 ms^a^	2.10 mV		
**Left Median Motor Nerve**								
Wrist-APB	3.10 ms	2.4 mV			3.42 ms	1.87 mV		
Antecubital fossa-Wrist	14.2 ms	0.56 mV	21.6 m/s	−76.7%	13.90 ms	0.58 mV	21.9 m/s	−69.0%
**Right Median Motor Nerve**								
Wrist-ABP	2.9 ms	9.2 mV			2.73 ms	9.5 mV		
Antecubital fossa-Wrist	9.5 ms	4.2 mV	37.1 m/s	−54.3%	8.44 ms	6.1 mV	42.0 m/s	−35.8%
**Left Ulnar Motor Nerve**								
Wrist-ADM	2.03 ms	11.1 mV			2.72 ms	12.4 mV		
Bellow elbow-Wrist	8.25 ms	3.8 mV	37.0 m/s	− 65.8%	9.02 ms	4.9 mV	36.5 m/s	− 60.5%
Above elbow-Bellow elbow	10.9 ms	3.2 mV	54.7 m/s	−15.8%	11.00 ms	4.8 mV	50.5 m/s	−2.0%
**Right Ulnar Motor Nerve**								
Wrist-ADM	2.20 ms	15.1 mV			2.00 ms	13.5 mV		
Bellow elbow-Wrist	8.20 ms	9.2 mV	64.5 m/s	−39.1%	5.85 ms	11.0 mV	58.4 m/s	−18.5%
Above elbow-Bellow elbow	6.0 ms	9.0 mV	59.1 m/s	−2.2%	7.69 ms	10.8 mV	55.4 m/s	−1.8%
**Left Tibial Motor Nerve**								
Ankle-AH	3.78 ms	17.2 mV			3.97 ms	16.2 mV		
Popliteal Fossa-Ankle	11.4 ms	9.1 mV	51.8 m/s	−47.1%	11.80 ms	10.0 mV	47.3 m/s	−38.3%
**Right Tibial Motor Nerve**								
Ankle-AH	4.62 ms	8.3 mV			5.89 ms	11.5 mV		
Popliteal Fossa-Ankle	14.2 ms	3.8 mV	40.7 m/s	−54.2%	14.90 ms	4.2 mV	43.3 m/s	−63.5%
**Left Deep Peroneal Motor Nerve**								
Ankle-EDB	3.82 ms	0.41 mV			6.52 ms	0.91 mV		
**Right Deep Peroneal Motor Nerve**								
Ankle-EDB	0.00 ms	0.00 mV			0.00 ms	0.00 mV		

Since the criteria for demyelinating polyneuropathy with conduction blocks were obtained, more proximal nerve segments and other proximal nerves were not studied

*APB* Abductor pollicis brevis, *ADM* Abductor digiti minimi, *AH* Abductor hallucis, *AMP* Amplitude, *CV* Conduction velocity, *DML* Distal motor latency, *EDB* Extensor digitorum brevis, *ms* Milisecond, *mV* Millivolt, *NP* Not performed

^a^DML normal adult mean values: 3.40 ± 0.80 ms [10]

**Table 2:** Sensory Nerve Conduction Studies Before and After Influenza A Infection

	Before H1N1 infection		After H1N1 infection (after 9 days)	
Nerve	AMP	CV	AMP	CV
**Left Median Sensory Nerve** (Wrist-Dig II)	5.6uV	56.0 m/s	17.6uV	47.8 m/s
**Right Median Sensory Nerve** (Wrist-Dig II)	34.0uV	47.9 m/s	26.1uV	55.0 m/s
**Left Superficial Radial Nerve** (Lateral Forearm-Anatomical Snuffbox)	20.1uV	67.9 m/s	38.4uV	64.9 m/s
**Right Superficial Radial Nerve** (Lateral Forearm-Anatomical Snuffbox)	0.0uV	0.0 m/s	0.0uV	0.0 m/s
**Left Ulnar Sensory Nerve** (Wrist-Dig V)	27.0uV	63.8 m/s	29.2uV	50.7 m/s
**Right Ulnar Sensory Nerve** (Wrist-Dig V)	20.0uV	55.0 m/s	28.8uV	62.8 m/s
**Left Superficial Peroneal Sensory Nerve**	6.0uV	60.0 m/s	7.1uV	51.7 m/s
**Right Superficial Peroneal Sensory Nerve**	0.0uV	0.0 m/s	0.0uV	0.0 m/s
**Left Sural Sensory Nerve**	7.4uV	47.9 m/s	7.5uV	40.2 m/s
**Right Sural Sensory Nerve**	4.1uV	38.6 m/s	5.3uV	36.2 m/s

*AMP* Amplitude, *CV* Conduction velocity, *Dig II* Second finger, *Dig V* Fifth finger, *ms* Milisecond, *uV* Microvolt

## Discussion/conclusion

Our patient presented a parainfectious hyperacute severe relapse of Lewis-Sumner Syndrome in the context of H1N1 virus pneumonia. The condition occurred so suddenly that it mimicked stroke. Antiviral treatment with oseltamivir sufficed to reverse the neuropathy and was extremely effective, without the need of immunomodulatory adjuvant therapy. After the 2009 influenza A pandemic it was evident that this virus is frequently associated with acute central and/or peripheral neurological signs and symptoms. Also, there are reports of acute relapses of CIDP after influenza vaccination [11, 12], as well as with other acute viral infections, such as hepatitis A, hepatitis B and hantavirus infections [2, 3]. In this instance, we believe there was an association between the active influenza A infection and hyperacute relapse of the LSS. Nevertheless, as far as we know, this is the first report of such a rapid relapse of LSS related to an active infection by H1N1 virus (or even due to other infectious agents), and stroke had to be ruled out because of its obvious implications in terms of pathogenesis, treatment and prognosis.

The modulation of host cell ion channel activity by viral proteins is being increasingly identified as an important virus–host interaction [13]. For instance, in the lung, the rate of sodium (Na+) absorption and chloride secretion controls the thickness of the fluid layer covering the respiratory surfaces. Influenza A virus has the ability to significantly inhibit Na + channels, altering the pulmonary fluid homeostasis, giving the virus ideal conditions to replicate [7, 13, 14]. Similarly, coronavirus S and E proteins also have shown to be capable of decreasing Na + channel activity [14]. Ion channels are fundamental regulators of action potential generation and signal transduction in excitable cells, such as neurons. In fact, multiple viral infections of the peripheral nervous system are thought to be expedited through the modulation of ion channels. For instance, varicella-zoster virus, a known cause of post-herpetic neuralgia, was shown to induce changes in Na + channels known to be associated with neuropathic pain [14], that supports the use of carbamazepine to treat this condition.

In nodopathies, electrical conduction blocks are caused by the inhibition of sodium and potassium channels at the Ranvier’s nodules. This is mediated by antibodies and resolves quickly after eliminating the antibodies from the circulation.

There are different possibilities as to the pathophysiology behind this case. Direct viral neuritis, especially in a patient with chronically damaged peripheral nerves, could be the cause. The fast recovery with oseltamivir, without the need for immunoglobulins, renders an immune mediated pathophysiology less likely. A third, interesting possibility is that in patients with some degree of neuronal sodium channel dysfunction, as in LSS patients, Influenza A could acutely aggravate this dysfunction causing hyperacute relapses and rapid recovery with viral elimination. The patient presented primary demyelinating neuropathy, as is evident from the facial nerve involvement, favoring either neuropaxia or functional transmission blockade as the most likely pathophysiologies, as observed in nodopathies. The rapid recovery with the antiviral agent, without IVIg, supports the possibility of channel dysfunction rather than an autoimmune response.

The world is now facing a new, more aggressive viral pandemic by coronavirus disease 2019 (Covid-19), and its consequences are still largely unknown. A report from China, were the outbreak of the virus SARS-CoV-2 was first identified, describes neurologic symptoms in 36.4% of the 214 hospitalized patients (even higher in patients more severely affected). Peripheral nervous system (PNS) involvement was noted in 8. 9% of the cases, including taste, smell and vision impairment, and, less frequently, nerve pain, and occurred early in the disease. However, nerve conduction studies were not performed and the duration of the PNS involvement was not reported [4]. It is still unknown whether this virus can trigger or exacerbate peripheral neuropathies, and efforts towards systematic and complete data collection on neurological manifestations should be made. On the other hand, although H1N1 has become a public health threat of global concern, there are no accurate data regarding the worldwide frequency of neurological complications related to this disease.

We intent to raise awareness about the possibility of atypical neurological presentations of these novel, highly infectious and dangerous respiratory virus, reinforcing the recommendation to perform diagnostic tests to identify them [5, 15, 16], as appropriate, in all patients with respiratory illness and any neurological signs/symptoms. We also believe that host’s cell ion channel dysfunctions represent an exciting and emerging field of virus–host interactions. We encourage more studies on the subject to allow a greater certainty about the suggested pathophysiological mechanism which ultimately may lead to potential ion channel drugs as new pharmacologically antiviral therapeutic strategies [14, 17].

## Data Availability

Data sharing is not applicable to this article as no datasets were generated or analysed during the current study.

## Abbreviations

LSS: Lewis-Sumner Syndrome
CIDP: Chronic Inflammatory Demyelinating Polyneuropathy
NCS/EMG: Electromyography with nerve conduction studies
iRODS: Rasch-built Overall Disability Scale
CT: Computer tomography
MRI: Magnetic resonance imaging
DML: Distal motor latencies
PNS: Peripheral nervous system
